# Does preoperative percutaneous nephrostomy insertion worsen upper-tract urothelial cancer oncological outcome? A retrospective single center study

**DOI:** 10.1186/s12894-019-0482-4

**Published:** 2019-06-07

**Authors:** Guan-Lin Huang, Hao-Lun Luo, Po-Hui Chiang

**Affiliations:** Department of Urology, Kaohsiung Chang Gung Memorial Hospital and Chang Gung University College of Medicine, 123, Ta-Pei Road, Niaosung, Kaohsiung, Taiwan

## Abstract

**Background:**

Physicians doubt percutaneous nephrostomy (PCN) insertion on cancer related hydronephrosis patients causes tumor seeding and worse cancer control. In this article, we attempted to determine if preoperative PCN alters cancer control in upper tract urothelial cancer (UTUC) patients.

**Methods:**

Retrospective analysis of UTUC patients in a single center from 2005 to 2015. Exclusion criteria included lymph node metastasis, and patients underwent perioperative adjuvant chemotherapy or radiotherapy. There were 664 patients in this analysis, with clinico-pathological data being collected retrospectively for Cox-regression statistical analysis. Outcomes were measured by local recurrence, distant metastasis and cancer-specific death with Kaplan-Meier curves.

**Results:**

There were respectively 25 and 639 UTUC cancers in the preoperative PCN and non-PCN insertion groups with mean follow-up duration of 37.9 and 48.6 months, respectively. The preoperative PCN group consisted of 17 patients (68%) with tumor located in the ureter, while the PCN-negative group included 236 patients (36%) with tumor located in the ureter being statistically significant. These two groups were comparable in gender, age, follow-up duration, tumor stage, and pathological features of the UTUC. As for the cancer control in the PCN group, 4(16%), 1(4%) and 1(4%) had local recurrence, distant metastasis and cancer-specific death respectively; in the non-PCN group, 101(15.8%), 96(15%) and 72(11.2%) exhibited local recurrence, distant metastasis and cancer-specific death respectively. Statistical analysis showed no difference in oncologic outcomes between these two groups.(*p* = 0.804, 0.201 and 0.254).

**Conclusions:**

Preoperative percutaneous nephrostomy on upper-tract urothelial cancer poses little risk on tumor seeding and could be considered as part of treatment strategy if renal function preservation is needed.

## Background

Hydronephrosis with compromised renal function is one manifestation of upper tract urothelial cancer [1]. Before definitive upper tract urothelial cancer diagnosis, percutaneous nephrostomy (PCN) may be undertaken for severe infective hydronephrosis or impending renal failure for temporary disease control. Although tumor seeding and invasion through the percutaneous nephrostomy tract has been reported on case reports [2, 3], there are no studies comparing locally or systemically driven cancer recurrence rates between preoperative PCN dwelling and non-PCN dwelling patients in preoperative upper-tract urothelial cancer patients. This study is the first study to analyze these two groups’ of patients in the best of our knowledge.

## Methods

We retrospectively collected upper-tract urothelial cancer patients receiving nephroureterectomy from 2005 to 2015 in single tertiary referring center. Procedures in the PCN group were initially intended for the preservation of deteriorating renal function or medical failure infective hydronephrosis with information of possible malignant tumor seeding through the PCN tract to patients. Interventional radiologist performed whole PCN group patients with Fr 10. nephrostomy tube or pigtail. Radical nephroureterectomy procedure cases were enrolled in the study for definite UTUC treatment. Peri-operative positive lymph nodes and distant organ metastasis cases were excluded due to poor prognosis and possible need of further adjuvant treatment; besides, neo-adjuvant or adjuvant candidates were also excluded for comparability of the disease course. Patient basic characteristics and pathological features were collected, such as age, gender, follow up duration, pathological stage, tumor figuration, tumor site, lymphovascular invasion, carcinoma in situ, multifocal, tumor necrosis, smoking and end stage renal disease. The follow-up protocol after nephroureterecotmy was as follows: 1.Computed tomography (CT) abdomen scanning every 3–6 months in the first 2 years and annually thereafter. 2. Cystoscopy every 3 months until 2 years shifted to every 6 months if negative finding. 3. Physical examination over the surgical wound and PCN tract during outpatient clinics visits, and CT scanning if suspicion of disease recurrence. Outcome measurements were classified as bladder recurrence, local recurrence, and distant metastasis. Follow-up cystoscopy showed merely bladder recurrence residual bladder cuffing recurrence was deemed as bladder recurrence. In PCN group, local recurrence was defined as disease recurrence over ipsilateral retroperitoneal space or PCN sites. In PCN negative group, local recurrence was defined as disease over the ipsilateral retroperitoneal space. Distant metastasis was defined as regional/distant lymph nodes metastasis or organ metastasis. We used Cox-regression statistical analysis on comparability and significance of tumor recurrence between PCN (+) and PCN (−) groups. Kaplan-Meier survival curve was applied for time to recurrence between these two groups.

## Results

In total, 25 patients in the PCN(+) group and 639 patients in the PCN(−) group had mean follow up duration of 37.9 and 48.6 months, respectively. The percentage of ureter tumor in the PCN(+) group(68%) was higher than the PCN(−) group(36%) with significant difference(*p* = 0.002). The median PCN dwelling time was 18 days [IOR: 5–26] Other basic characteristics such as gender, age, tumor stage, tumor grade, lymphovascular invasion, were collected with no significant difference between these two groups (Table 1.)

**Table 1:** Patient demography and oncological outcome

	Preop PCN(+)	Preop PCN(−)	
	*n* = 25	*n* = 639	
Gender(Male/Female)	11/14	366/273	0.189
Age(years)	70.8 ± 9.1	67.17 ± 10.8	0.098
Follow up duration	37.9 ± 27.8	48.6 ± 31.4	0.119
pT stage			0.353
pT0	6	181	
pT1	10	174	
pT2	5	122	
pT3	3	154	
pT4	1	8	
Papillary	18	506	0.378
Tumor location			
RP tumor	4	264	0.011
U tumor	17	236	0.002
RP + U tumor	4	138	0.790
High grade	23	578	0.796
LVI	6	97	0.232
CIS	7	226	0.449
Multifocal	9	189	0.491
TN	7	184	0.931
Smoking	5	65	0.116
ESRD	5	132	0.937
Prior bladder Ca	6	147	0.908
Oncological result			
Bladder recurrence	8	201	0.983
Local recurrence	4	101	0.804
Distant metastasis	1	96	0.201
Cancer specific death	1	72	0.254

*RP tumor* renal pelvis tumor, *U tumor* ureter tumor, *LVI* lymphovascular invasion

*CIS* carcinoma in situ, *TN* tumor necrosis, *ESRD* end stage renal disease

As for the cancer control in the PCN group, 4(16%), 1(4%) and 1(4%) had local recurrence, distant metastasis and cancer specific death respectively; in the non-PCN group, 101(15.8%), 96(15%) and 72(11.2%) had local recurrence, distant metastasis and cancer specific death respectively. Statistical analysis showed no cancer control difference between these two groups.(*p* = 0.804, 0.201 and 0.254)(Table 1.)

**Fig. 1:**
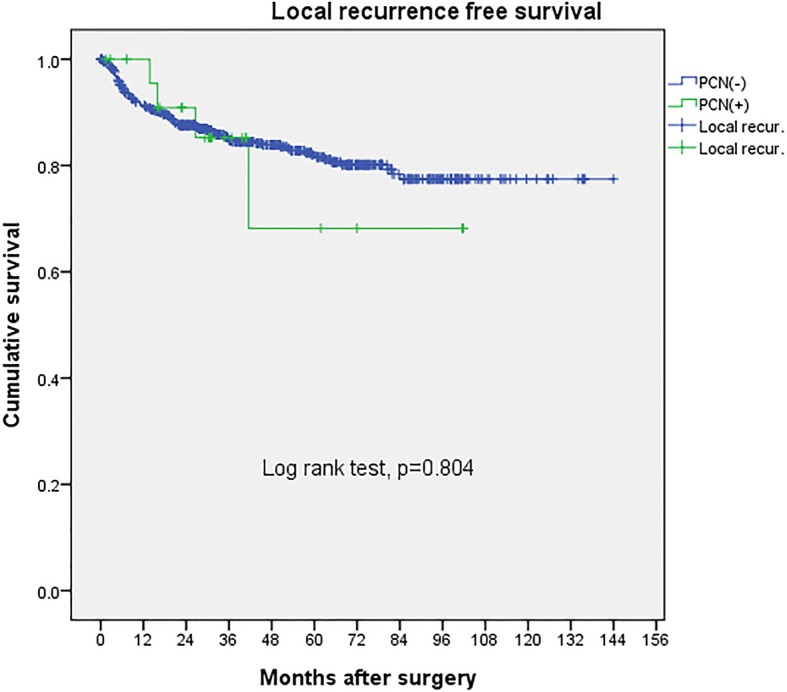
Upper tract urothelial cancer local recurrence free survival after PCN

**Fig. 2:**
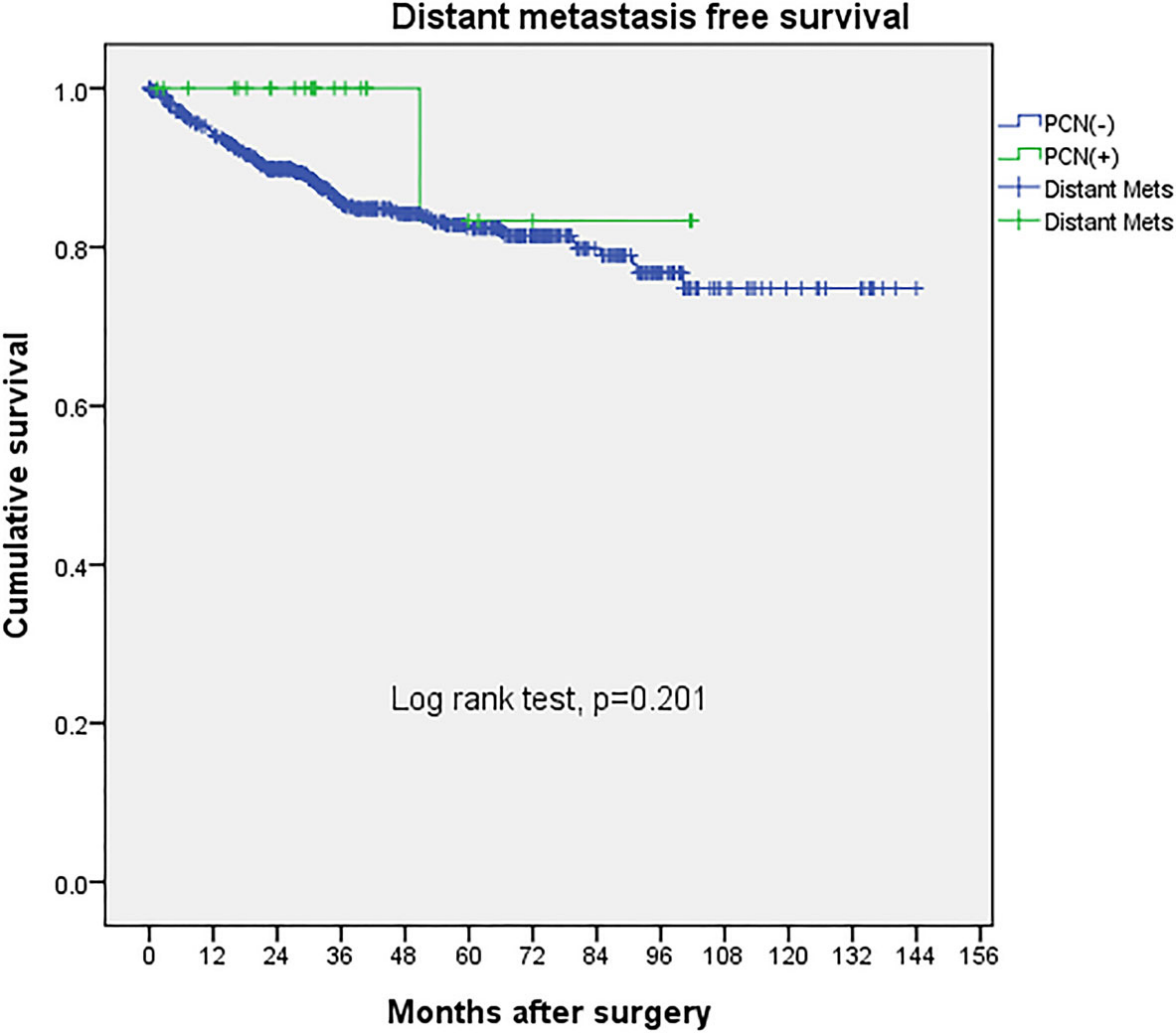
Upper tract urothelial cancer distant metastasis free survival after PCN

In the PCN group, no patients were found to have skin metastasis or PCN tract tumor invasion and the local recurrence pattern was over the ipsilateral retroperitoneal space, which is similar to PCN negative group. Besides, time to being local-recurrence free and time to being distant metastasis free in Kaplan-Meier survival curve showed no difference between these two groups.(*p* = 0.804 & 0.201)(Fig 1. & Fig 2.)

## Discussion

Tumor seeding after the percutaneous nephrostomy insertion has been reported in case reports but no study has evaluated if PCN procedure alter the risk of tumor seeding or cancer control [2–4]. In our study, the demographic and clinic-pathological data were similar between these two groups except for distribution of tumor location (17(68%) and 236(52%) ureter tumors in the PCN and non-PCN groups respectively, *p* = 0.002). It is reasonable that ureteral tumors are often associated with more event of hydronephrosis and sometimes external drainage was sometimes inevitable. Besides, relieving the post-renal obstruction may be considered earlier than urothelial cancer management when presenting as severe renal function impairment. After standard radical nephroureterectomy, comparable local recurrence (PCN group vs non-PCN group: 4/25 vs 101/639, 16% vs 15.8%, *p* = 0.804) and distant metastasis rate(PCN group vs non-PCN group: 1/25 vs 96/639, 4% vs 15%, *p* = 0.201) between these two groups were noted. Furthermore, there was no tumor dissemination along the PCN tract or skin metastasis observed in our study; hence, the risk of the worse oncological outcome related to the PCN procedure should not be over-emphasized especially for patients with need of salvaging residual renal function.

Table 2 Summarized the previous case reports of tumor seeding through the PCN tract [3–9]. Synchronous or metachronous urothelial cancer in upper urinary tract and bladder were the risk factors for tumor seeding [3–9]. Percutaneous endoscopic resection of the renal pelvis tumor seems another circumstance for tumor seeding [3–5, 8, 9]. However, Goel et al. reported long-term oncological outcomes of upper tract urothelial cancer status post percutaneous endoscopic resection, and concluded risk of tract seeding was low on low grade tumor [10]. Palou et al. also found percutaneous resection of superficial UTUC with good long term cancer outcome [11]. Roupret et al. retrospectively collected 24 patients receiving the percutaneous endoscopic resection of the tumor and reported 5-year disease-specific and tumor-free survival rates were 79.5 and 68%. There was none track seeding in the study as well [12]. Serrano et al. reviewed articles in Medline regarding the cancer disseminating in the PCN tracts and concluded that risk of tumor seeding is very low, and there were still other explanation rather than PCN tract manipulation, like blood stream or lymphatic metastasis [13]. Through the PCN tract, percutaneous resection of upper tract urothelial cancer was an alternative option for preservation renal function with comparable long-term cancer control in low-grade and limited tumor size patients. Meanwhile, risk for the tumor seeding through the PCN tract should be informed but downplayed [10–12, 14, 15]. In addition, Kiss et al. mentioned that internal drainage within urinary tract may increase the risk or urothelial recurrence [16]. According to the reviewed literature, the external drainage carried low risk of recurrence and should be considered for relieving urinary obstruction.

**Table 2:** Case reports of tumor seeding through PCN track

Authors	Sex, M/F	Age, years	Concomitant with bladder cancer?	Tumor site	Histology	PCN function	Definite NUXBCE?	PCN dwelling time	Recurrence site	Time from PCN to recurrence
Sharma et al. 1994 [5]	M	56	Yes	RP, U	UC, high grade	PET	Yes	N.R.	Nephrectomy scar site	8 months
Huang et al. 1995 [6]	F	80	Yes	U	UC, high grade	Relief of obstruction, AP diagnosis	Yes	30 days	Nephrostomy track	1 month
Sengupta and Harewood,1998 [7]	M	78	Yes	RP	UC, high grade	Relief of obstruction	Yes	N.R.	Nephrostomy track	9 months
Yamada et al. 2002 [4]	M	63	Yes	RP	UC, low grade(G2)	PET	Yes	14 days	Nephrostomy track	3 months
Treuthardt et al. 2004 [8]	M	61	Yes	RP	UC, high grade(G2)	PET	No	6 weeks	Nephrostomy track	12 months
Wang et al. 2004 [9]	F	63	Yes	RP, U	UC, high grade(G3)	Relief of obstruction	Yes	7 days	Nephrostomy track	3 months
	M	52	No	RP	SCC, poor differentiated	PET	Yes	10 days	Nephrostomy track	4 months
Sorokin et al. 2013 [3]	M	76	Yes	RP	UC, high grade	PET	No	2 months	Nephrostomy track	5 months

*UC* urothelial cancer, *N.R.* not reported, *SCC* squamous cell carcinoma, *PCN* percutaneous nephrostomy, *NUXBCE* nephroureterectomy with bladder cuff excision, *PET* percutaneous endoscopic treatment, *AP* antegrade pyelography, *RP* renal pelvis, *U* ureter

In our study, 25 preoperative PCN patients were initially performed for relieving urinary obstruction and clinical diagnosis instead of percutaneous endoscopic manipulation; hence, the risk of tumor seeding and altered oncological outcomes was minimal after standard nephroureterectomy. In addition, the evidences of neoadjuvant chemotherapy are getting more and more and the oncologic result is inspiring [17, 18]; however, impaired renal function is relatively contraindicated for cisplatin-based chemotherapy and such situation is not uncommon in clinical practice especially for UTUC patients [19, 20]. The PCN drainage may preserve residual renal function for UTUC patients with hydronephrosis and can be considered as a treatment strategy before neoadjuvant chemotherapy.

### Limitation

The article is retrospective with relatively small-sized populations with short PCN dwelling time(Median: 18 days, IQR: 5–26). The prognostic effect of tumor site can not be fully assessed due to such small cohort by independent risk model. Further multi-center experience should be considered. Though the baseline asymmetry of ureteral tumor case between these two groups may cause of some bias, it reflect the real-world situation because that PCN was often used for preserving the residual function. The major size of PCN is eight to ten French in size and this study can further tell where if large size of PCN cause tumor seeding or not. However, this is the only study comparing PCN-related tumor seeding and oncological outcome, as we know.

## Conclusion

Preoperative percutaneous nephrostomy on upper-tract urothelial cancer poses little risk on tumor seeding and could be considered as part of treatment strategy if renal function preservation is needed.

## Data Availability

The datasets used and/or analyzed during the current study available from the corresponding author on reasonable request.

## Abbreviations

AP: antegrade pyelography
CIS: carcinoma in situ
CT: computed tomography
ESRD: end stage renal disease
LVI: lymphovascular invasion
NUXBCE: nephroureterectomy with bladder cuff excision
PCN: percutaneous nephrostomy
PET: percutaneous endoscopic treatment
RP tumor: renal pelvis tumor
RP: renal pelvis
SCC: squamous cell carcinoma
TN: tumor necrosis
TURBT: transurethral resection of bladder tumor
U tumor: ureter tumor
U: ureter
UC: urothelial cancer
UTUC: upper tract urothelial cancer
